# Redirecting Cell Fate During *in vitro* Embryogenesis: Phytoglobins as Molecular Switches

**DOI:** 10.3389/fpls.2018.01477

**Published:** 2018-10-09

**Authors:** Mohamed Elhiti, Shuanglong Huang, Mohamed M. Mira, Robert D. Hill, Claudio Stasolla

**Affiliations:** Department of Plant Science, University of Manitoba, Winnipeg, MB, Canada

**Keywords:** emrbyogenesis, phytoglobin, cell death, emrbyogenic tissue, nitric oxide

## Regulation of somatic-embryogenic transition by PGBS

During *in vitro* embryogenesis, the somatic embryogenic transition is a crucial step requiring a de-differentiation event in which information relative to somatic development is abrogated and an overall novel cellular reprogramming/reorganization initiated (Dudits et al., 1991). This switch, culminating in embryogenic competence and involving profound changes in chromatic structure, transcription, translation, and metabolism (Feher et al., 2003), is triggered by several factors, the most important of which are stress and growth regulators.

Stress is an inherent component experienced by explants during dissection and when cultured under physiologically suboptimal conditions. Within limits, stress promotes gene expression and metabolic changes linked to the induction of cellular dedifferentiation, and often sufficient to initiate the embryogenic pathway (Grosset et al., 1990; Pasternak et al., 2002). A well acknowledged stress-induced signal molecule is nitric oxide (NO), which participates in the promotion of cell division and embryogenic cell formation in alfalfa (Otvos et al., 2005).

Together with stress, plant growth regulators, especially auxin, have been linked to the somatic-embryogenic transition. Inclusion of different types of auxins in the culture medium is routinely used to trigger cellular dedifferentiation in both gymnosperms and angiosperms (Huang et al., 2016). An often cited example on the requirement of auxin for this event is the carrot culture system where embryo development from single cells is initiated by addition of the synthetic auxin 2,4-D (Nomura et al., 1995). Besides promoting cell division in the explant, auxins have been directly or indirectly linked to changes in gene expression, protein synthesis and turnover and chromatin reorganization necessary for the initiation of the embryogenic path (Feher et al., 2003).

Phytoglobins (Pgbs) are key molecules linking stress, NO, auxin metabolism and the acquisition of embryogenic competence. As well as acting as modulators of biotic and abiotic stress responses (Hill, 2012; Hill et al., 2013; Stasolla and Hill, 2017) by scavenging NO (Berger et al., 2018), Pgbs have been documented to influence the somatic embryogenic transition during Arabidopsis somatic embryogenesis. Specifically, suppression of *Pgb2* encourages the formation of embryogenic cells and thus the number of embryos generated from the cotyledons of the zygotic embryos used as explants (Elhiti et al., 2013). The authors proposed a model in which a reduction in *Pgb2* level elevates the amount of NO which down-regulates the expression of MYC2, a stress-related transcription factor inhibiting the synthesis of auxin. By relieving the inhibition on auxin synthesis, as well as by redirecting auxin flow through a relocation of PIN1, a required step for the induction of dedifferentiation related genes (Su et al., 2009), suppression of *Pgb2* favored embryogenic competence (Elhiti et al., 2013). These events can be replicated by targeting *Pgb2* in the cytoplasm (Godee et al., 2017). A further elaboration of this model, reinforcing the link between Pgb and stress response factors in the somatic-embryogenic transition includes the suppression of MYC2 by jasmonic acid (JA) in cells enriched in NO as a result of *Pgb2* suppression (Mira et al., 2017). A fundamental concept emerging from these studies is that the level of *Pgbs* in the cells is a determining factor in the redirection of the fate of a somatic cell toward an embryogenic pathway; this redirection seems to employ components shared in stress responses.

## Shaping the embryo body: regulation of programmed cell death (PCD) by PGBS

Together with division and differentiation, PCD is a conserved developmental process manifested during plant embryogenesis and required to dismantle the suspensor and degrade subordinate embryos in gymnosperms seeds (review in Huang et al., 2016). Execution of PCD is also an integral component of *in vitro* embryogenesis and was first described in spruce (Filonova et al., 2002). In this system, somatic embryos are produced from proembeyogenic masses (PEMs) grouped into three cellular aggregates (PEM I-III). While PEM I are formed by cytoplasmic cells subtended by a single suspensor-like cell, other PEMs are more elaborated, with PEM III consisting of many suspensor-like cells radiating from a cluster of cytoplasmic cells. Removal of plant growth regulators is needed to reshape PEM III into somatic embryos through massive execution of PCD, which is a required event (Filonova et al., 2002). An experimental inhibition of the death program through manipulation of the culture medium is indeed sufficient to prevent the formation of the embryos from PEM III (Bozhkov et al., 2002).

The requirement of PCD for embryo development is not restricted to somatic embryogenesis, but also observed during microspore-derived embryogenesis; an indirect process often initiated by the stress-inducible formation of multicellular structures (MCSs), which further differentiate into embryo-like structures (ELSs; Touraev et al., 1997). In this system two waves of PCD occur. The first wave during MCS formation eliminates the tapetal cell layer and this step is linked to the redirection of cell development toward the embryogenic fate (Wang et al., 1999; Varnier et al., 2009). The second wave of PCD is visible during the differentiation of MCSs (composed by two distinct cell domains derived from the generative cell and vegetative cell) into ELSs. The targeted elimination of the suspensor-like generative domain facilitates the development of the vegetative domain into ELSs (Maraschin et al., 2005). It is apparent that controlled execution of PCD is an obligatory step in many *in vitro* embryogenic systems, and therefore the identification of components triggering or suppressing the death program could be exploited to control embryo development.

A well-established component influencing the cellular death program is Pgb. During different types of stress, including hypoxia or water stress, the presence of Pgbs in specific cells plays a protective role. Cells lacking Pgbs die as a result of the imposed stress through mechanisms mediated by ethylene and reactive oxygen species (ROS), while those expressing *Pgbs* survive (Nomura et al., 1995; Mira et al., 2016a,b). Manifestation of the death program resulting from the reduced expression of *Pgb* also occurs during somatic embryogenesis. In maize, suppression of *ZmPgb1.1* or *ZmPgb1.2* induces PCD through an elevation of cellular NO contributing to the release of Zn^2+^ from methallothioneins. The cell-specific accumulation of Zn^2+^ triggers a mitogen-activate protein kinase (MAPK) cascade resulting in the production of ROS and the subsequence activation of PCD (Huang et al., 2014). Despite the similar mechanistic model of the two Pgbs, the effect of their suppression on embryogenesis is different: that is, down-regulation of *ZmPgb1.2* increases the number of somatic embryos, while down-regulation of *ZmPgb1.1* reduces the embryogenic output. This is due to the distinct expression domains of the two *Pgbs*. The expression of *ZmPgb1.2* is restricted to the cells anchoring the immature somatic embryos to the embryogenic tissue; thus removal of these cells by PCD as a result of *ZmPgb1.2* suppression releases the immature embryos allowing them to develop further at high frequency. Conversely, *ZmPgb1.1* is expressed in many embryonic cells and their dismantling by PCD, when *ZmPgb1.1* is repressed, leads to embryo abortion (Huang et al., 2014). Further studies on the same system also demonstrated the involvement of growth regulators in the Pgb regulation of the death/survival fate. Kapoor et al. (2018) showed that embryonic cells suppressing either *ZmPgb1.1* or *ZmPgb1.2* were depleted in abscisic acid, causing an activation of ethylene synthesis and response linked to the over-production of ROS and PCD. This model was substantiated by pharmacological treatments showing that manipulations of ABA and/or ethylene affected the Pgb regulation of embryogenesis.

## Concluding remarks

**Figure 1:**
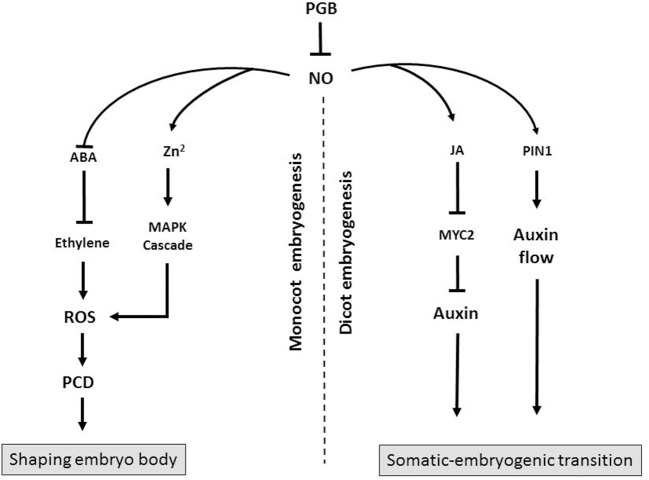
Diagram showing the possible mode of action of Pgbs during monocot and dicot embryogenesis. In monocot embryogenesis Pgb suppresses NO which promotes ROS-mediated PCD through a release of Zn^2+^ triggering a mitogen-activate protein kinase (MAPK) cascade. Alternatively, NO suppresses ABA, an inhibitor of ethylene. Execution of PCD, as a result of Pgb suppression, shapes the embryos and influences the embryogenic output. In dicots, Pgb suppresses NO, an inducer of MYC2, which suppresses auxin synthesis. Nitric oxide also influences auxin flow through PIN1. Production of auxin, as a result of *Pgb* suppression, favors the somatic-embryogenic transition.

While it is unarguable that Pgbs act as “molecular switches” by influencing cell fate during two crucial steps of *in vitro* embryogenesis, two key points are worth mentioning relative to the somatic-embryogenic transition and the execution of the death program shaping the body of the embryos. First, it is apparent that is impossible to describe Pgb action through a single model consistent between monocots and dicots. As described above the action of Pgbs during Arabidopsis somatic embryogenesis is executed by auxin and the PIN1 auxin transport, while in maize Pgbs act by the induction of PCD. Both actions, however, are mediated by the NO scavenging properties of Pgbs (Figure 1). Second, while different Pgbs might exercise the same functions, their overall effect on *in vitro* embryogenesis depends on their localization domain, a concept best exemplified in maize. The fact that plants have different *Pgbs*, and that their promoter regions contain diverse elements (Hill et al., 2013), supports the fact that they might operate in different domains characterized by specific physiological environments. Thus, characterizing these physiological environments and more specifically upstream components modulating the expression of *Pgbs* should be a priority in plant embryogenesis.

## Author contributions

ME, SH and MM contributed equally in writing different sections of the manuscript. RH and CS contributed with new idea and interpretations of data.

### Conflict of interest statement

The authors declare that the research was conducted in the absence of any commercial or financial relationships that could be construed as a potential conflict of interest.
